# Impact of leaflet thrombosis on hemodynamics and clinical outcomes after bioprosthetic aortic valve replacement: A meta‐analysis

**DOI:** 10.1002/clc.23331

**Published:** 2020-01-20

**Authors:** Zixin Tian, Tiejun Li, Shumei Ma

**Affiliations:** ^1^ Department of Cardiology Shengjing Hospital of China Medical University Shengyang Liaoning Province People's Republic of China

**Keywords:** aortic stenosis, bioprosthetic aortic valve, surgical aortic valve replacement, thrombosis, transcatheter aortic valve replacement

## Abstract

**Background:**

Leaflet thrombosis (LT, also called cusp thrombosis) detected by multidetector computed tomography (MDCT) is common in bioprosthetic aortic valve replacement (bAVR). However, it remains contradictory whether MDCT‐defined LT following bAVR is associated with hemodynamic deterioration and stroke. Thus, we performed the first meta‐analysis to assess hemodynamic outcomes and updated the latest researches on the clinical outcomes of MDCT‐defined LT after bAVR.

**Hypothesis:**

MDCT‐defined LT might be associated with worse hemodynamic and clinical outcomes after bAVR.

**Method:**

MEDLINE, EMBASE, Cochrane Library, and http://clinicaltrial.gov were searched from inception to 15th April 2019. The fix‐effect model was utilized to calculate odds ratio (OR) and 95% confidence interval (CI). The primary outcomes were hemodynamic stability indexes, including mean pressure gradient (MPG), left ventricular ejection fraction (LVEF), paravalvular leak (PVL), and clinical heart failure. The secondary endpoints were major adverse cardiovascular and cerebrovascular events (MACCEs), which consisted of myocardial infarction, all‐cause death, stroke, and transient ischemic attack (TIA).

**Results:**

Twelve studies with 4820 patients were included. The total prevalence of MDCT‐defined LT was 9.7%. MDCT‐defined LT was associated with a significantly increased risk of MPG (inverse variance 0.43, 95% CI: [0.30, 0.57]), MACCEs (OR 2.43, 95% CI: [1.45, 4.06]), stroke (OR 1.79, 95% CI: [1.03, 3.11]), and TIA (OR 4.09, 95% CI: [1.59, 10.54]). There were no differences for other outcomes.

**Conclusions:**

MDCT‐defined LT after bAVR is associated with increased MPG and increased risk of adverse cerebrovascular events, including TIA and stroke. While LVEF, PVL, and clinical heart failure were similar between patient with and without LT.

## 1 INTRODUCTION

Surgical aortic valve replacement (SAVR) or transcatheter aortic valve replacement (TAVR) have been recommended in both American and European guidelines for patients with symptomatic severe aortic stenosis.^1^, ^2^ Bioprosthetic aortic valve is an implanted device made of non‐synthetic origin to replace severe dysfunctional aortic valve during SAVR or TAVR. Recently, reports have shown that TAVR is suitable not only for surgical‐prohibitive or high‐risk patients,^3^, ^4^, ^5^, ^6^ but also for intermediate and low‐risk patients,^7^, ^8^, ^9^, ^10^ and therefore, the number of patients undergoing TAVR is expected to be up to 270 000 in Northern‐America and Europe annually.^11^ Bioprosthetic aortic valve replacement (bAVR) during TAVR is fast becoming a key treatment for relieving symptomatic severe aortic valve stenosis. Leaflet thrombosis (LT, also called cusp thrombosis) is a common complication in bAVR with a prevalence of about 7% to 15% detected on multi‐detector computed tomography (MDCT).^12^, ^13^, ^14^ MDCT has been proven to be a more sensitive method for detection of (LT), which is characterized by hypoattenuated leaflet thickening (HALT) and a reduction in leaflet motion (RELM).^15^ Some studies report contradictory findings about the association of adverse clinical events with MDCT‐defined LT in bAVR.^16^, ^17^, ^18^, ^19^, ^20^ On the one hand, the impact of MDCT‐defined LT on hemodynamic indexes remained unidentified. On the other hand, it remains controversial whether MDCT‐defined LT following bAVR is associated with stroke. Two previous meta‐analysises^21^, ^22^ with 5 and 6 included studies respectively conclude inconsistent conclusions on this topic. As new evidence emerges in recent years, a new meta‐analysis is needed to reassess this problem. Therefore, we performed a meta‐analysis and systematic review of the incidence, and the hemodynamic and clinical outcomes of MDCT‐defined LT following TAVR or SAVR to explore whether MDCT‐defined LT induced deteriorating hemodynamics and adverse cerebrovascular events.

## 2 METHODS

This systematic review was based on the Preferred Reporting Items for Systematic Reviews and Meta‐Analyses (PRISMA) statement. We searched the MEDLINE, EMBASE, and Cochrane Library databases for electronically published papers, and http://clinicaltrials.gov for unpublished literature or ongoing trials up to 15th April 2019 to evaluate the effect of MDCT‐defined LT in bAVR on hemodynamics and clinical outcomes. The search strategy utilized terms synonymous with the TAVR, SAVR, bioprosthetic aortic valve, and thrombosis, including “transcatheter aortic valve replacement,” “transcatheter aortic valve implantation,” “ bioprosthetic aortic valve,” “computed tomography,” and “thrombosis.” A manual search for all references of included studies was performed simultaneously.

The inclusion criteria were as follows: (a) report of LT in TAVR or SAVR patients, (b) post‐TAVR or post‐SAVR MDCT or four‐dimensional computed tomography (4D CT) imaging performed during follow‐up, (c) clinical outcomes reported between patients with and without MDCT‐defined LT, and (d) retrospective or prospective cohort studies. Studies were excluded if: (a) there was no control group, (b) diagnosis of LT was based on other imaging modalities, (c) only the abstract was published, and (d) there was less than a 6‐month follow‐up. We chose the newest papers with the largest population if there were several studies reporting on the same cohort of patients. In this study, LT was specifically defined as evidence of HALT or RELM of more than 50% in at least one leaflet on MDCT.

Two investigators performed the meta‐analysis independently, including study selection, risk‐of‐bias assessment, data extraction, and analysis. If there were any discrepancies, they were resolved by consensus. The risk of bias was assessed according to the Newcastle Ottawa Scale.^23^

The primary outcome of this study was hemodynamic stability, as measured by mean pressure gradient (MPG), left ventricular ejection fraction (LVEF), clinical heart failure, and more than moderate paravalvular leak (PVL) during follow‐up. Major adverse cardiovascular and cerebrovascular event (MACCE) was the secondary endpoint, which comprised myocardial infarction (MI), all‐cause death, and stroke or transient ischemic attack (TIA). Stroke was defined as either neurological dysfunction of >24 hours with neuroimaging evidence or diagnosis by neurologists, while TIA was defined as transient neurological dysfunction of <24 hours without neuroimaging evidence of stroke or diagnosis by neurologists. MI was defined in the presence of at least two of the following manifestations: ischemic clinical symptoms, increased cardiac biomarker or electrocardiogram changes, or diagnosis by cardiologists. Clinical heart failure was specifically defined as rehospitalization for heart failure or new‐onset heart failure diagnosed by cardiologists. A worse than moderate PVL was defined according to the Valve Academic Research Consortium‐2 criteria.^24^

Statistical analysis was performed using Review Manager 5.3 (The Nordic Cochrane Centre, The Cochrane Collaboration, Copenhagen, Denmark) and Stata version 14.0 (StataCorp, College Station, Texas). Fixed effect Mantel‐Haenszel or Peto models were utilized to calculate odds ratios (OR) for binary variables and continuous variables were analyzed using a fixed‐effect inverse variance (IV) model to determine standard mean differences with 95% confidence intervals (CI). Statistical heterogeneity was rated as high, moderate, or low based on *I* ^2^ values of 75%, 50%, and 25%, respectively. Egger's linear regression tests were performed to assess publication bias. A *P*‐value of <.05 was considered significant.

## 3 RESULTS

**Figure 1:**
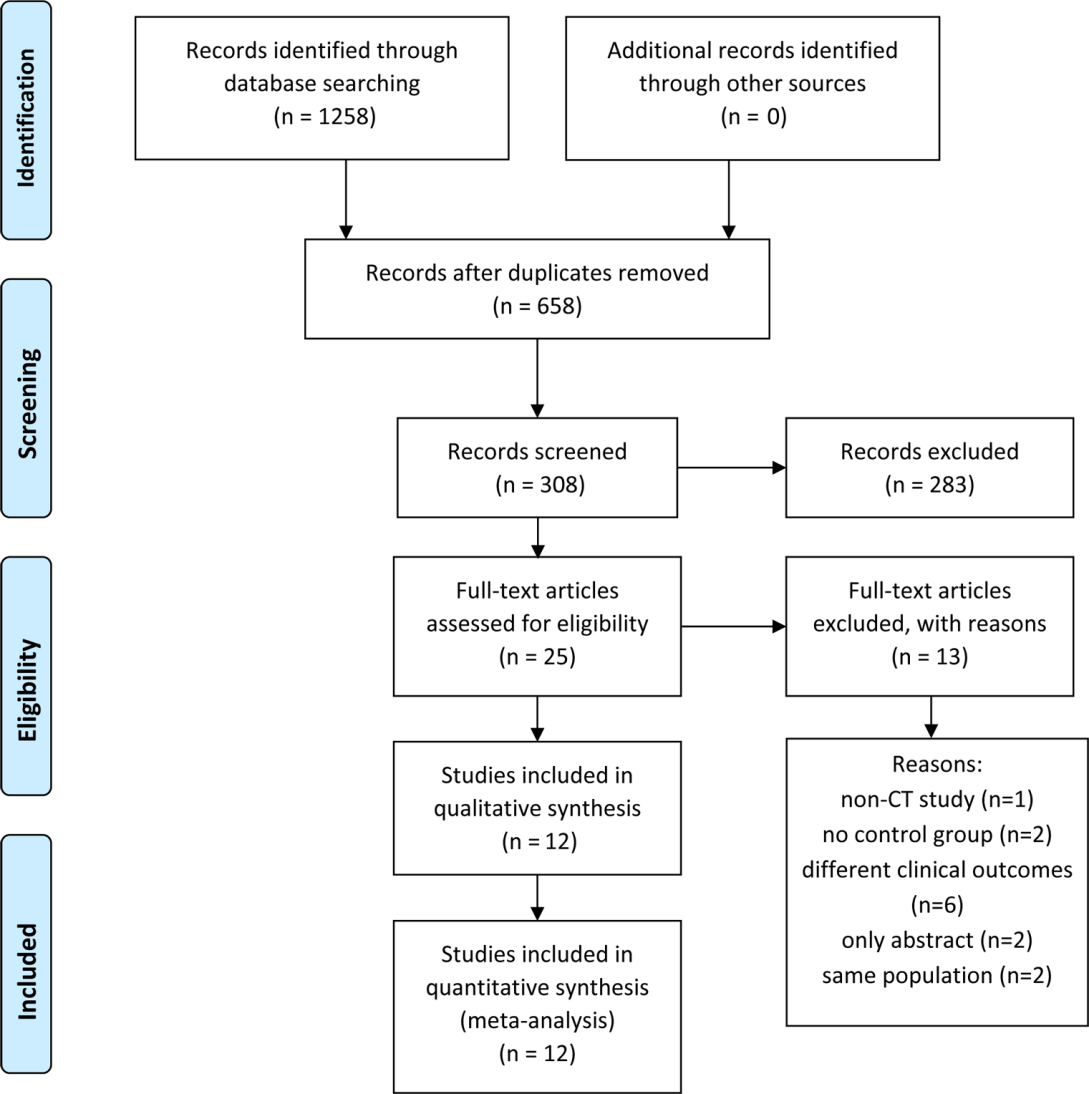
Study flow chart. CT, computed tomography

The search strategies yielded a total of 25 citations and 12 studies met the criteria (Figure 1). These 12 studies included reported results from the PORTICO IDE trial^23^ and the combined results of RESOLVE and SAVORY registries.^16^ The other 10 studies were single‐center observational cohort studies. In total, 4820 patients were included in this analysis, and 4636 patients received TAVR and 184 patients received SAVR. The demographic details are presented in Table 1. Overall, 452 patients (9.7%) were identified with evidence of LT on MDCT. Table S1 shows the quality assessment characteristics of the included studies.

**Table 1:** Demographic characteristics of included studies

Study	Sample (n)	LT (n)	Age (mean)	Male (%)	Smoker (%)	HTN (%)	DM (%)	AF (%)	MI (%)	PCI or CABG (n)	Stroke or TIA (%)	Thrombotic history (%)	STS score (mean)	EuroScore II (mean)	LVEF (%)	MPG (%)
Tang et al. (2019)^18^	287	26	83.2/80.9	65/51	0/2	80/84	23/32	38/38	15/7	38/55	12/15	4/2	3.9/4.5	—	56/57	33.6/39.1
Yanagisawa et al. (2019)^35^	485	45	85/85	27/31	—	—	36/26	13/23	—	18/24	41	7/9	—	—	—	—
Franzone et al. (2018)^12^	1396	10	79.7/82.1	50/51	—	90/85	10/26	—	—	30/14	10/11	—	5.3/6.0	—	57.2/53.8	45.7/42.2
Ruile et al. (2018)^20^	754	120	83/82	37/47	18/19	88/91	25/28	—	11/16	9/11	13/14	—	—	12.4/12.3	49/49.5	—
Marwan et al. (2018)^19^	78	18	81/81	67/42	—	73/75	46/21	20/48	7/6	40/30	20/2	—	—	—	53/53	42/39
Vollema et al. (2017)^15^	128	16	81/81	50/52	25/48	78/77	78/69	25/30	19/21	—	38/19	0/4	—	23/23	50.9/49.8	41.6/42
Chakravarty et al. (2017)^16^	890	106	82/78.9	60/56	—	83/87	21/25	16/30	—	—	8/8	—	—	—	55.5/57.9	44.6/44.2
Hansson et al. (2016)^14^	405	28	83	60/56	—	—	18/23	21/48	29/24	21/51	22/32	—	—	—	—	—
Pache et al. (2015)^13^	156	16	83.2/82.1	38/46	—	—	13/23	38/42	0/13	6.3/20	—	6/19	4.7/4.5	14.7/11.4	50/53.3	—
Makkar et al. (2015)^33^	55	22	86.3/82.1	36/55	—	95/85	55/52	45/45	9/12	46/78	31/24	—	—	—	—	—
Basra et al. (2018)^17^	101	32	73/77	—	—	81/83	31/26	25/42	—	—	6/1	3/1	7/6	—	45.9/52.5	—
Jimenez et al. (2019)^43^	85	13	81.8/82.1	39/32	8/17	77/81	8/28	8/25	—	—	—	—	4.3/4.3	3.7/5.5	56.3/55.9	—

*Note*: Data are reported as LT/Non‐LT.

Abbreviations: AF, atrial fibrillation; CABG, coronary artery bypass graft; COPD = chronic obstructive pulmonary disease; DM, diabetes mellitus; HTN, hypertension; LT, leaflet thrombosis; LVEF, left ventricular ejection fraction; MI, myocardial infarction; MPG, mean pressure gradient; PCI, percutaneous coronary intervention; TIA, transient ischemic attack; STS, Society of Thoracic Surgeons.

**Figure 2:**
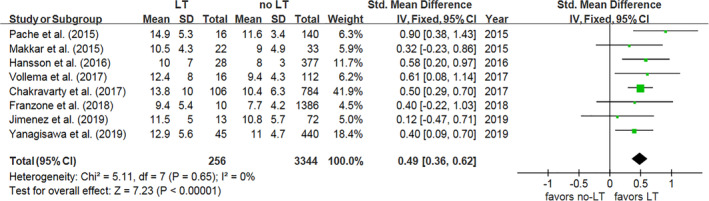
Forest plot for mean pressure gradient. IV, inverse variance; LT, leaflet thrombosis; Std, standard; 95% CI, 95% confidence interval

Eight studies compared MPG in patients with and without LT during follow‐up, and the MPG was significantly higher in patients with LT compared to those without LT (IV 0.49, 95% CI: [0.36, 0.62], *P* < .00001, *I* ^2^ = 0%, *P* = .65) (Figure 2). However, there were no differences in LVEF (%) (IV −0.17, 95% CI: [−0.36, 0.01], *P* = .06, *I* ^2^ = 47%, *P* = .15) (Figure S1), clinical heart failure (OR 0.66, 95% CI: [0.31, 1.42], *P* = .29, *I* ^2^ = 0%, *P* = .99) (Figure S2) or rate of more than a moderate PVL (OR 1.05, 95% CI: [0.34, 3.21], *P* = .93, *I* ^2^ = 0%, *P* = .52) (Figure S3).

Four studies reported the incidence of MACCE with a summary OR of 2.43 (95%CI: [1.45, 4.06], *P* = .0007), demonstrating an increased risk of MACCE in patients with evidence of LT on MDCT (Figure S4). The statistical heterogeneity between studies was low (*I* ^2^ = 0%, *P* = .44).

**Figure 3:**
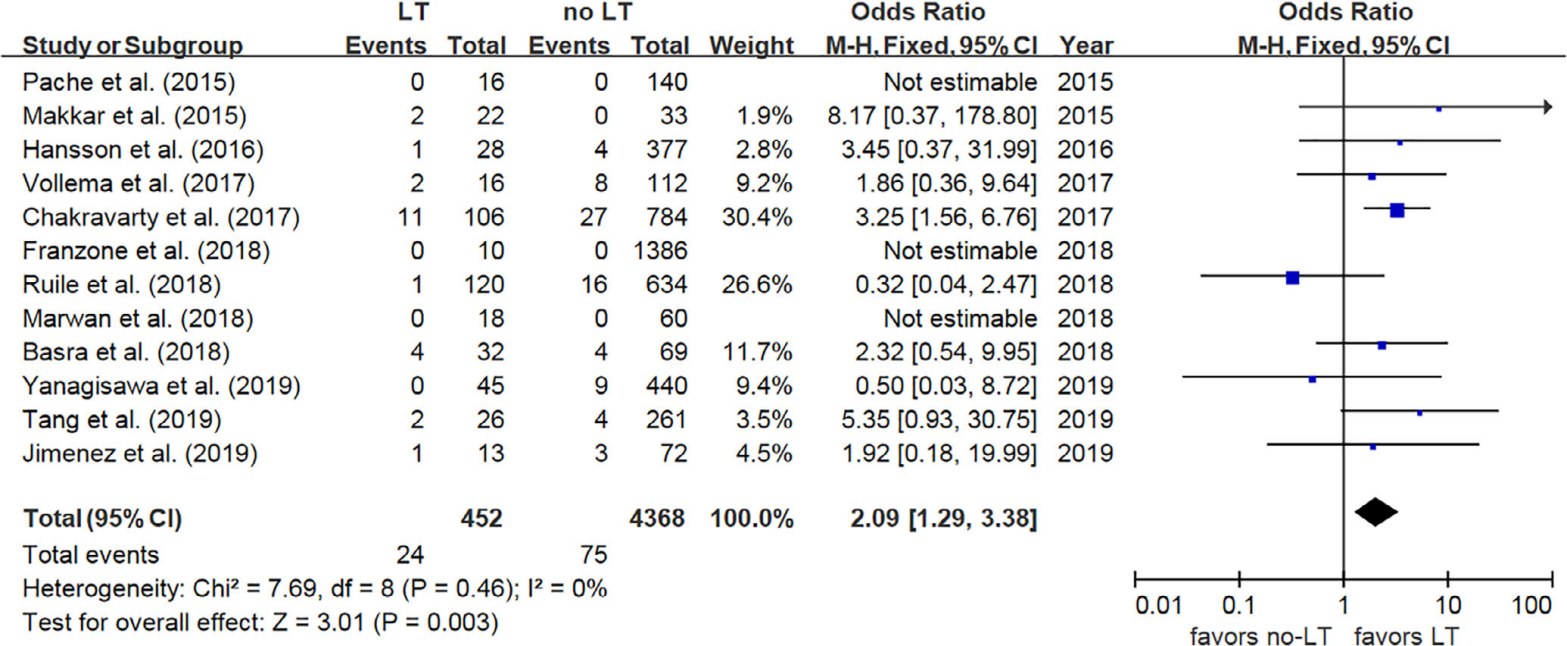
Forest plot for cerebrovascular events. LT, leaflet thrombosis; M‐H, Mantel‐Haenszel model; 95% CI, 95% confidence interval

**Figure 4:**
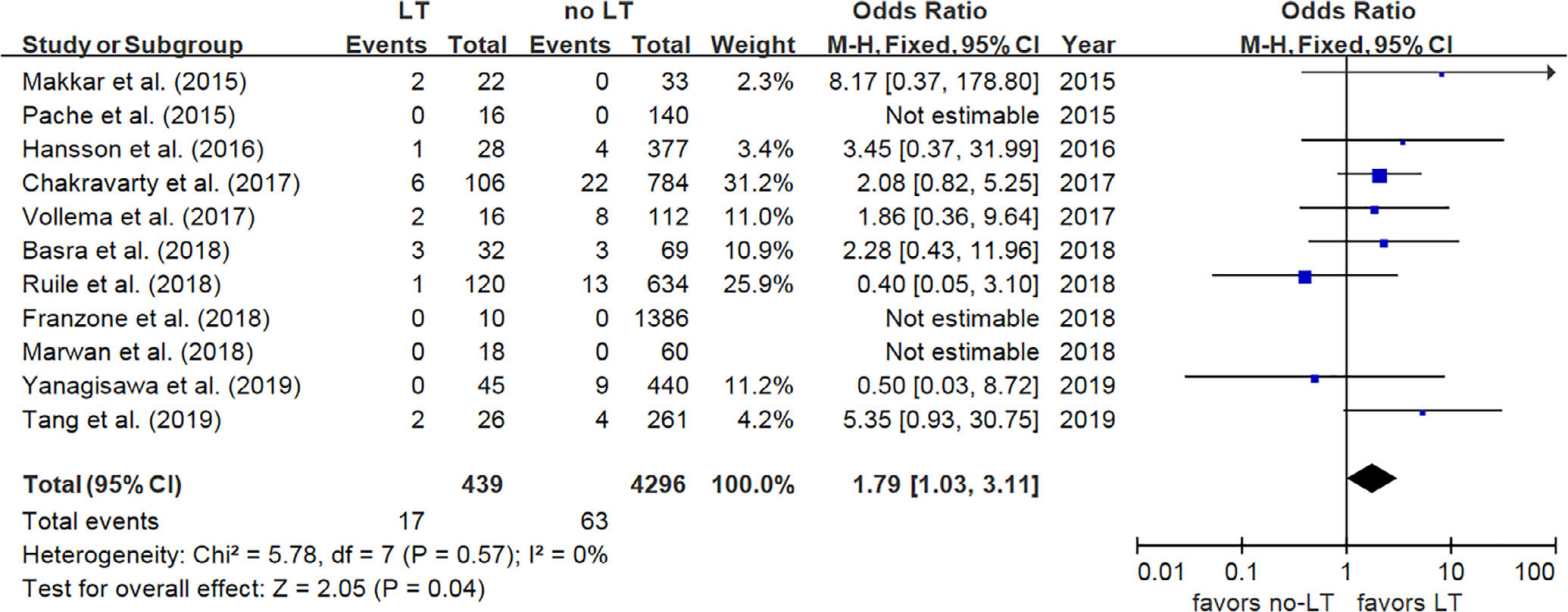
Forest plot for stroke. LT, leaflet thrombosis; M‐H, Mantel‐Haenszel model; 95% CI, 95% confidence interval

The incidence of adverse cerebrovascular events in LT and no‐LT patients was reported in 12 studies, and the combined results demonstrated an increased risk of adverse cerebrovascular events in LT patients (OR 2.09, 95% CI: [1.29, 3.38], *P* = .003, *I* ^2^ = 0%, *P* = .46) (Figure 3). Similar findings were found for stroke (OR 1.79, 95% CI: [1.03, 3.11], *P* = .04, *I* ^2^ = 0%, *P* = .57) (Figure 4) and TIA (OR 4.21, 95% CI: [1.58, 11.23], *P* = .004, *I* ^2^ = 49%, *P* = .16) (Figure S5). However, there was no significant differences for MI (OR 2.85, 95% CI: [0.72, 11.36], *P* = .14, *I* ^2^ = 0%, *P* = .54) (Figure S6) or all‐cause death (OR 0.65, 95% CI: [0.42, 1.01], *P* = .06, *I* ^2^ = 0%, *P* = .67) (Figure S7) in patients with and without LT.

There was no publication bias regarding clinical outcomes (*P* = .284, Figure S8) or hemodynamic outcomes (*P* = .618, Figure S9).

## 4 DISCUSSION

To the best of our knowledge, this is the first meta‐analysis to investigate the hemodynamic effects in LT patients and we update the largest dataset to date to investigate adverse clinical outcomes in patients with LT detected on MDCT. The most obvious finding to emerge from this analysis is that MDCT‐defined LT affects the MPG, but not LVEF, which causes clinical heart failure. Moreover, we have clarified the clinical outcomes of MDCT‐detected LT, and we show that the incidence of stroke was significantly higher in LT patients, and this result is found without statistical heterogeneity (OR 1.79, 95% CI: [1.03, 3.11], *P* = .04). On the one hand, these results indicate that the hemodynamic changes caused by LT do not influence the short‐ or medium‐term cardiac function, but may affect long‐term valve durability. On the other hand, the correlation between LT and stroke demonstrates that more research regarding a tailored antithrombosis regime is warranted to improve the quality of life of patients.

Bioprosthetic aortic valve thrombosis is associated with endothelial injury, hemodynamic stasis, altered LVEF, and atrial fibrillation (AF).^25^, ^26^ A strong relationship between hemodynamic stasis and the formation of LT has been reported in the literature.^27^, ^28^, ^29^ In this study, MDCT‐defined LT is found to be associated with increased MPG; however, we do not observe any association between LT and decreased LVEF, increased risk of clinical heart failure, or all‐cause death. This finding is consistent with that of Douglas et al. who performed a 3‐year follow‐up study of patients after TAVR and revealed that LT detected on transthoracic echocardiography (TTE) was associated with increased MPG, which was associated with increased risk of death and reintervention.^30^ A possible explanation for the all‐cause death discrepancy might be the specific populations selected for MDCT and the relatively shorter follow‐up periods in the eligible studies, as well as the exclusion of patients with an unexplained cause of death before MDCT and patients with renal dysfunction. We cannot ignore the moderate statistical heterogeneity of LVEF in our study because very few studies were available. Thus, we strongly encourage further research about the impact of MDCT‐defined LT on hemodynamic stability and its correlation with clinical outcomes.

Lethal stroke occurs in about 7% of patients in the first year after TAVR.^31^ Nevertheless, the rate of new‐onset stroke increases to two‐thirds of patients following TAVR if neuroimaging is used.^32^ A previous meta‐analysis demonstrated that MDCT‐defined LT was associated with increased risk of cerebrovascular events and TIA, but not stroke,^21^ while another meta‐analysis showed that LT detected by echocardiography or MDCT was associated with increased risk of stroke.^22^ While we find similar results for TIA, we find that there is an increased risk of stroke in patients with MDCT‐defined LT. Rashid et al. performed three studies^16^, ^33^, ^34^ that reported on the incidence of TIA, but only two studies^16^, ^35^ are included in our study with moderate bias, because Yanagisawa et al. reported on the same patient cohort in 2016 and did not report on the incidence of TIA in their most recent study. We believe that the heterogeneity of TIA in our study comes from the small sample size, in that the *I* ^2^ became 6% when we added the incidence of TIA reported by Yanagisawa et al. in 2016 (Figure S10). Regarding stroke, our study shows a trend towards a significantly increased risk of stroke in patients with LT (OR 1.79, 95% CI: [1.03, 3.11], *P* = .04). However, we interpret this result carefully because the method used to diagnose stroke varied between studies. Moreover, the rates of AF vary between included studies and evidences showed that one‐third of ischemic strokes attribute to AF^36^. But it seems influent little on the result because previous studies find an impact of new‐onset but not pre‐existing AF on the early stroke in TAVR.^37^, ^38^ And the incidence of stroke are still higher in MDCT‐defined LT in the included studies where rates of AF are equal in the LT and non‐LT group^33^, ^35^ during 1‐year follow‐up. Large‐scale, standardized, MRI‐determined stroke studies, such as the TICTAVI (NCT02817789) and AUREA (NCT01642134) trials, are warranted to further elucidate whether LT detected by MDCT indicates the occurrence of stroke.

Given that it is possible that MDCT‐defined LT is related to hemodynamic deterioration and MACCEs, this is an important issue for physicians. Several reports have shown that HALT and RELM regress after anticoagulation.^39^, ^40^ The initiation of anticoagulation depends on the discretion of physicians. Anticoagulation seems to reduce the occurrence of subclinical LT more effectively than antiplatelets. Despite the RESOLVE and SAVORY registries mentioned in this article, the FRANCE‐TAVI registry experiment, which investigated subclinical thrombotic events in 12 804 patients, showed that LT rates were significantly lower in patients who received an anticoagulation regimen.^42^ Jimenez et al.^43^ observed that lack of oral anticoagulant therapy at discharge was an independent predictor of MDCT‐defined LT in patients following TAVR. The mechanism behind the lower occurrence of LT in patients administered anticoagulation agents might be that adenosine diphosphate‐induced platelet reactivity was not significantly associated with the occurrence of HALT.^44^ However, anticoagulation therapy may not be appropriate for all patients following bAVR. The global study comparing a rivaroxaban‐based antithrombotic strategy to an antiplatelet‐based strategy after GALILEO trial (NCT02556203), which compared results in two groups: an experimental group who received rivaroxaban and aspirin for the first 3 months and then rivaroxaban indefinitely, and a control group who received a single antiplatelet for the first 3 months and then aspirin indefinitely, was prematurely terminated due to increased all‐cause death, bleeding, and thromboembolic events. This indicated that it may be necessary to determine the effectiveness of an antithrombotic regimen after fully evaluating the risk of thrombosis and bleeding. There are other ongoing registry or random control trial like ENVISAGE‐TAVI AF trial (NCT02943785), ATLANTIS trial (NCT02664649), etc., exploring the optimal anticoagulation regimen in patients following TAVR. Until the results of these trials are revealed, the safety and effectiveness of preventive anticoagulation in patients following bAVR remain unknown.

Our research has several strengths. First, to our best knowledge, this is the first meta‐analysis evaluating hemodynamic changes in patients with MDCT‐defined LT after bAVR. Second, our report involves the largest sample size to date (4636 patients), and this means that our report has stronger statistical power regarding the association between stroke and MDCT‐defined LT compared to previous meta‐analyses. Third, most of the results in our study involved no heterogeneity or low heterogeneity, except for LVEF because very few studies were available. There were also some limitations to our research. First, most of the included studies were observational cohort studies, therefore, it is inevitable that hidden biases may have influenced our results. Second, the interval from bAVR to MDCT varied between studies, and this may have impacted the prevalence of LT. Although the timing of the first MDCT differed in the included studies (ranging from 3 days to 1 year), spontaneous regression of LT is rare and usually follows months of anticoagulant administration, according to the research. Third, we only assessed MPG, PLV, and LVEF changes during follow‐up from only a few studies. More studies are warranted to investigate more hemodynamic indexes, such as peak pressure gradient, effective orifice area, etc. Fourth, although hemodynamic indexes were assessed by TTE in all of included studies, interval between TTE and bAVR, lost to follow‐up and methodology of measurement varied between studies, which might contribute to the clinical heterogeneity. Finally, for hard endpoints, event numbers are relatively low and studies are not using the same definitions, this is a caveat. Meta‐regression or subgroup analysis is not feasible due to the limited number of included studies. Further work should be performed with more evidence available.

In conclusion, MDCT‐defined LT following TAVR/SAVR is associated with changed hemodynamic stability and a significantly increased risk of MACCEs, especially adverse cerebrovascular events, including TIA and stroke. Therefore, we recommend that MDCT is performed where possible to detect high‐risk patients for LT, such as patients with the morbidity of AF and CHA2DS2‐VASc≥2, history of thrombolism, low LVEF, the elderly, etc. Further studies are required to explore whether MDCT‐detected LT influences other hemodynamic indexes, even valve durability in the long term, and whether the adverse cerebrovascular events can be adequately prevented with the use of anticoagulants.

## Supporting information


**Figure S1** Forest plot for LVEF (%) displaying summary odds ratio (OR) and 95% confidence intervals (CI). LT, leaflet thrombosis; Std., standard; IV, inverse variance; 95% CI, 95% confidence interval

**Figure S2** Forest plot for clinical heart failure displaying summary odds ratio (OR) and 95% confidence intervals (CI). LT, leaflet thrombosis; M‐H, Mantel‐Haenszel model; 95% CI, 95% confidence interval

**Figure S3** Forest plot for more than moderate paravascular leak displaying summary odds ratio (OR) and 95% confidence intervals (CI).
LT, leaflet thrombosis; M‐H, Mantel‐Haenszel model; 95% CI, 95% confidence interval

**Figure S4** Forest plot for major adverse cardiovascular and adverse cerebrovascular events. LT, leaflet thrombosis; M‐H, Mantel‐Haenszel model; 95% CI, 95% confidence interval

**Figure S5** Forest plot for transient ischemic attack. LT, leaflet thrombosis; M‐H, Mantel‐Haenszel model; 95% CI, 95% confidence interval

**Figure S6** Forest plot for more than myocardial infarction displaying summary odds ratio (OR) and 95% confidence intervals (CI).
LT, leaflet thrombosis; M‐H, Mantel‐Haenszel model; 95% CI, 95% confidence interval

**Figure S7** Forest plot for all‐cause death
LT, leaflet thrombosis; M‐H, Mantel‐Haenszel model; 95% CI, 95% confidence interval

**Figure S8** Egger test for major adverse cardiovascular and cerebrovascular events, including stroke, transient ischemic attack, myocardial infarction, moderate paravalvular leak, and all‐cause death

**Figure S9** Egger test for hemodynamic measures, including mean pressure gradient, left ventricular ejection fraction and clinical heart failure

**Figure S10** Forest plot for transient ischemic attack including Yanasawa et al. (2016). LT, leaflet thrombosis; M‐H, Mantel‐Haenszel model; 95% CI, 95% confidence interval

**Table S1** Quality assessment of included studies
LT, leaflet thrombosis
