# Supplementary figures and images for: Impact of leaflet thrombosis on hemodynamics and clinical outcomes after bioprosthetic aortic valve replacement: A meta‐analysis
Source: Clin Cardiol. 2020 Jan 20;43(5):468–74. doi: 10.1002/clc.23331 (PMC7244303; doi:10.1002/clc.23331)

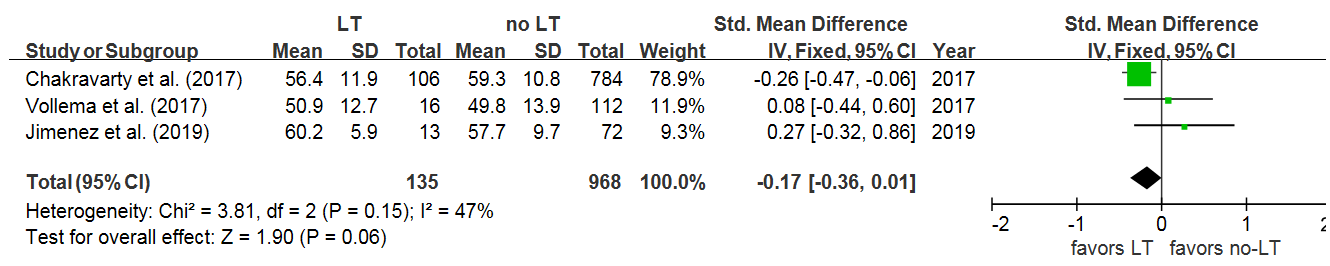

Supplement: Supplementary file 1 — Figure S1 Forest plot for LVEF (%) displaying summary odds ratio (OR) and 95% confidence intervals (CI). LT, leaflet thrombosis; Std., standard; IV, inverse variance; 95% CI, 95% confidence interval [file CLC-43-468-s001.tif]

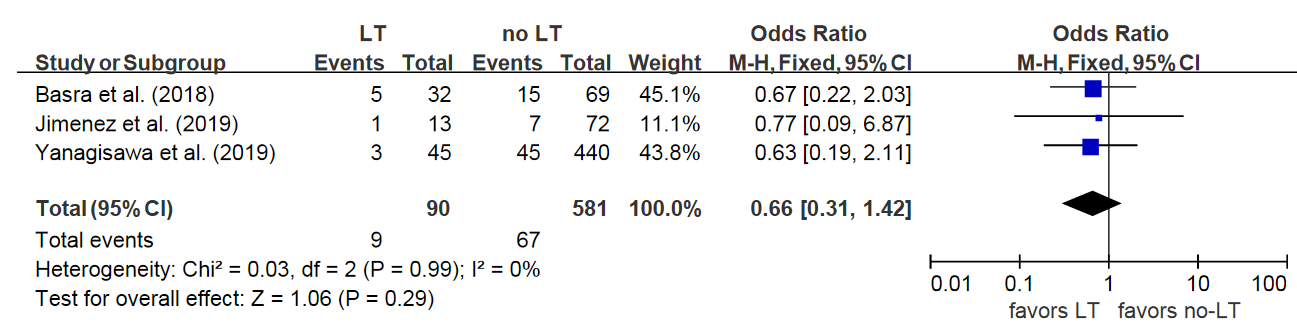

Supplement: Supplementary file 2 — Figure S2 Forest plot for clinical heart failure displaying summary odds ratio (OR) and 95% confidence intervals (CI). LT, leaflet thrombosis; M‐H, Mantel‐Haenszel model; 95% CI, 95% confidence interval [file CLC-43-468-s002.tif]

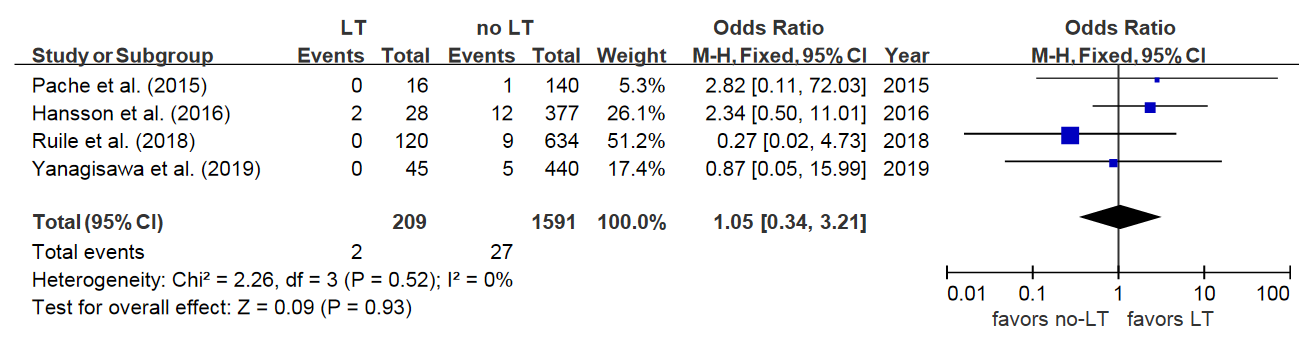

Supplement: Supplementary file 3 — Figure S3 Forest plot for more than moderate paravascular leak displaying summary odds ratio (OR) and 95% confidence intervals (CI). LT, leaflet thrombosis; M‐H, Mantel‐Haenszel model; 95% CI, 95% confidence interval [file CLC-43-468-s003.tif]

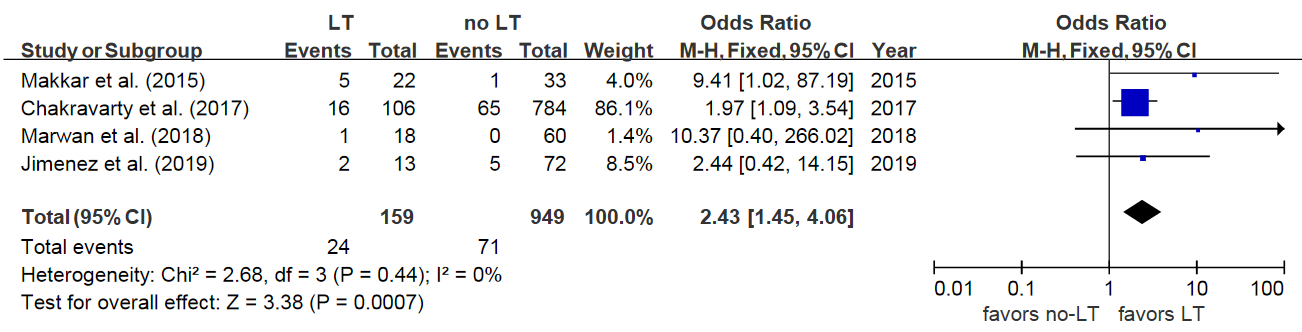

Supplement: Supplementary file 4 — Figure S4 Forest plot for major adverse cardiovascular and adverse cerebrovascular events. LT, leaflet thrombosis; M‐H, Mantel‐Haenszel model; 95% CI, 95% confidence interval [file CLC-43-468-s004.tif]

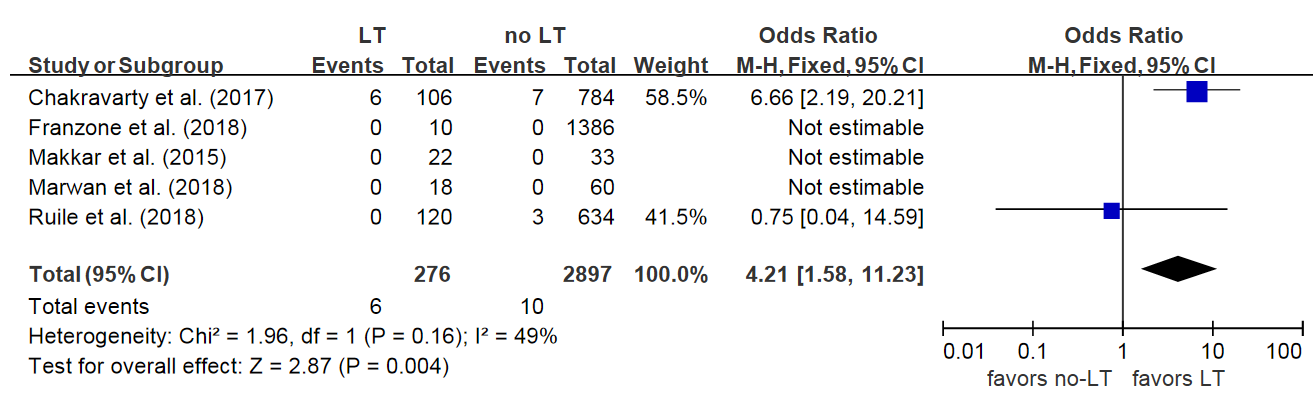

Supplement: Supplementary file 5 — Figure S5 Forest plot for transient ischemic attack. LT, leaflet thrombosis; M‐H, Mantel‐Haenszel model; 95% CI, 95% confidence interval [file CLC-43-468-s005.tif]

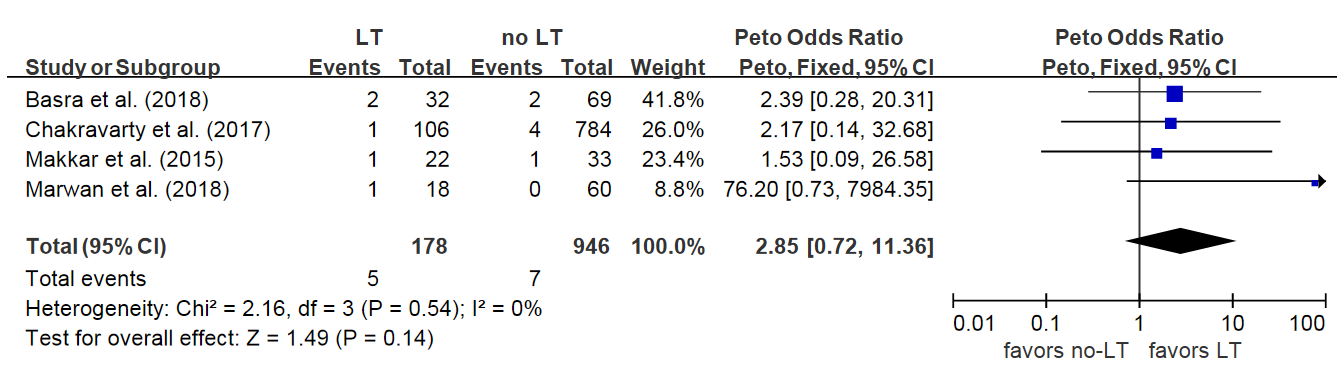

Supplement: Supplementary file 6 — Figure S6 Forest plot for more than myocardial infarction displaying summary odds ratio (OR) and 95% confidence intervals (CI). LT, leaflet thrombosis; M‐H, Mantel‐Haenszel model; 95% CI, 95% confidence interval [file CLC-43-468-s006.tif]

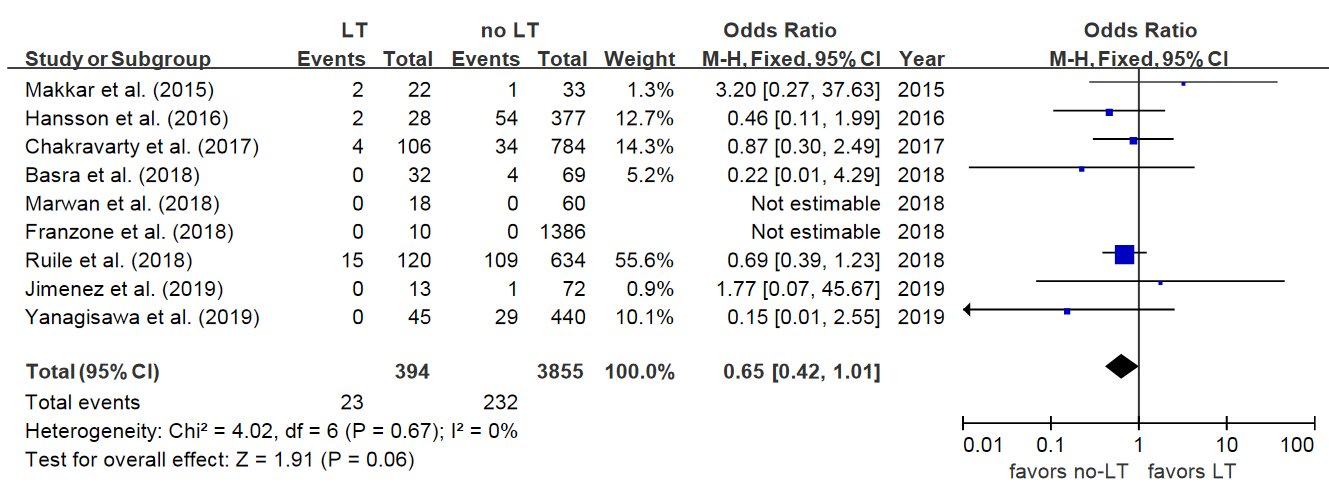

Supplement: Supplementary file 7 — Figure S7 Forest plot for all‐cause death LT, leaflet thrombosis; M‐H, Mantel‐Haenszel model; 95% CI, 95% confidence interval [file CLC-43-468-s007.tif]

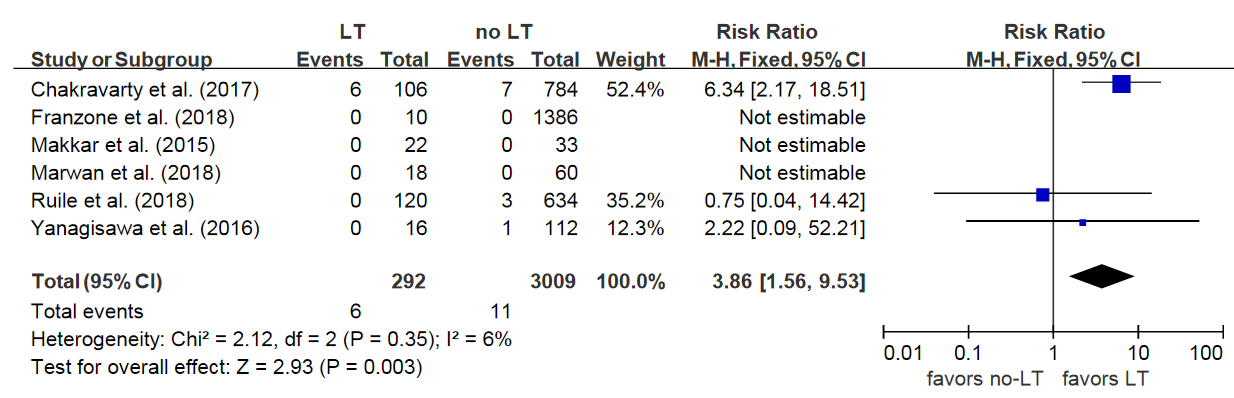

Supplement: Supplementary file 10 — Figure S10 Forest plot for transient ischemic attack including Yanasawa et al. (2016). LT, leaflet thrombosis; M‐H, Mantel‐Haenszel model; 95% CI, 95% confidence interval [file CLC-43-468-s010.tif]
